# Monte Carlo dosimetry of a novel Yttrium‐90 disc source for episcleral brachytherapy

**DOI:** 10.1002/acm2.14140

**Published:** 2023-09-14

**Authors:** Xiangyun Chang, Lyu Huang, Jian Liu, Yijian Cao, Jenghwa Chang

**Affiliations:** ^1^ MS in Medical Physics Program, Department of Physics and Astronomy Hofstra University Hempstead New York USA; ^2^ Radiation Medicine Northwell Health Lake Success New York USA; ^3^ Radiation Oncology Rhode Island Hospital Providence Rhode Island USA; ^4^ Radiation Medicine Zucker School of Medicine at Hofstra/Northwell Lake Success New York USA

**Keywords:** HDR brachytherapy, Monte Carlo, Y‐90

## Abstract

**Purpose:**

To calculate the dose distribution using Monte Carlo simulations for a novel high‐dose‐rate Yttrium‐90 (Y‐90) disc source recently developed for episcleral brachytherapy and provide a lookup table for treatment planning.

**Methods:**

Monte Carlo simulations were performed to calculate the in‐water dose distribution of the Y‐90 disc source using the “GATE”, a software based on the “Geant4” Monte Carlo simulation toolkit developed by the international OpenGATE collaboration. The geometry of this novel beta source, its capsule, and the surrounding water medium were accurately modeled in the simulation input files. The standard Y‐90 element beta spectrum from ICRU 72 was used, and the physics processes for beta and photon interactions with matters were all included. The dose distribution of this Y‐90 disc source was measured in a separate study using Gafchromic EBT‐3 films and the results were reported elsewhere. To match the setup of the experiment, a Gafchromic EBT‐3 film was also included in the simulation geometry. The simulated dose profiles were exported from the 3D dose distribution results and compared with the measured dose profiles. Transverse dose profiles at different distances from the seed surface were also obtained to study the lateral coverage of the source.

**Results:**

The measured percent depth dose (PDD) curves along the central axis perpendicular to the surface of the Y‐90 disc were constructed from the experimental and simulated data, and normalized to the reference point at 1 mm from the source capsule. Both PDD curves agreed well up to 4 mm from the source surface (maximum difference ± 10%) but deviated from each other beyond 4 mm. The deviation might be caused by the increased measurement uncertainty in the low‐dose region. The dose rate at the reference point calculated from the Monte Carlo simulation was 1.09 cGy/mCi‐s and agreed very well with the measured dose rate of 1.05 cGy/mCi‐s. If the 80% isodose line is selected as the lateral coverage, the lateral dose coverage is maximal (∼4.5 mm) at the plane next to the source surface, and gradually decreases with the increasing distance, approaching 3.5 mm when the plane is 5 mm from the 6‐mm diameter source surface.

**Conclusion:**

Monte Carlo simulations were successfully performed to confirm the measured PDD curve of the novel Y‐90 disc source. This simulation work laid a solid foundation for characterizing the full dosimetry parameters of this source for episcleral brachytherapy applications.

## 1 INTRODUCTION

**FIGURE 1:**
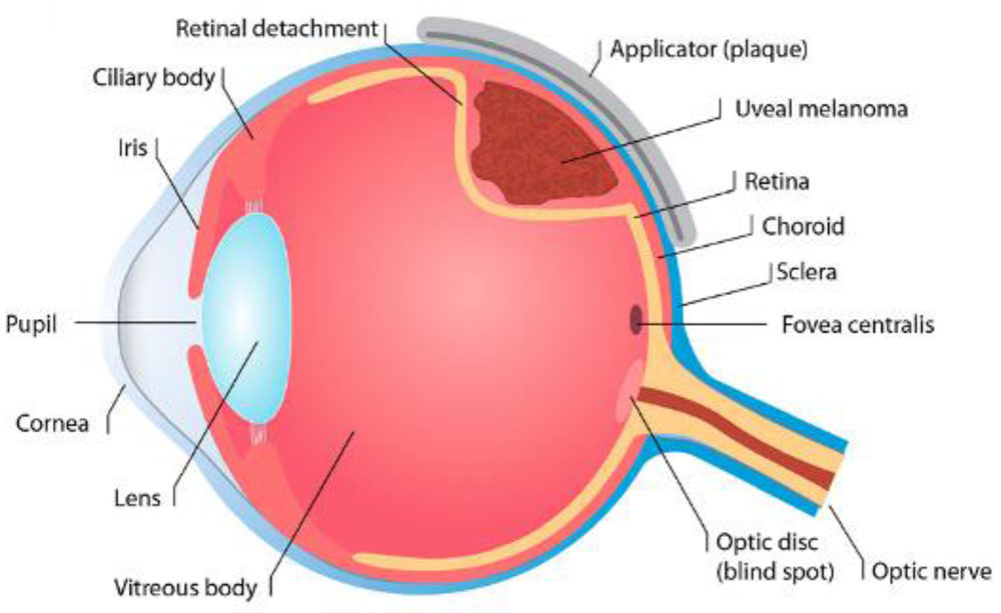
Ru‐106/Rh‐106 eye applicator in an artist's representation of eye anatomy (image courtesy Eckert & Ziegler BEBIG GmbH).^4^

Eye plaque brachytherapy (EPB) has been widely used as an effective treatment for uveal melanoma.^1^, ^2^, ^3^ In this treatment, evenly distributed radioactive seeds are fixed through an applicator (plaque) (Figure 1),^4^ in the space between the eyeball and ophthalmic cavity in contact with the uveal melanoma. The plaque then stays in that location for a specified period of time so that the prescribed radiation dose can be delivered to the tumor for treatment. It has been found that there is no difference in outcome for medium‐sized uveal melanoma patients treated with plaque brachytherapy or enucleation.^1^ Therefore, more patients choose brachytherapy when it is recommended as an option.^5^

Table 1 summarizes the isotopes that could be used for eye plaque brachytherapy. Traditionally, the seeds used for plaque brachytherapy are Ru‐106/Rh‐106 seeds or I‐125 (or much less often Pd‐103), despite the availability of many other radioisotopes for treatment.^4^ Ru‐106/Rh‐106 is commonly used for EPB in Europe and Asia. It is a beta‐ray emitter with a peak energy of 3.54 MeV, an average energy of 1.42 MeV, and a half‐life of 373.6 days. Ru‐106/Rh‐106 plaques can be prescribed for tumors with less than 5 mm thickness.^6^ Due to the interaction properties of beta‐ray with matters, Ru‐106/Rh‐106 has a sharp dose fall‐off, which is preferred for sparing normal tissues. I‐125 is a gamma‐ray emitter with a peak energy of 35.5 keV and a half‐life of 59.5 days. Despite the much lower energy of the photons than that of Ru‐106/Rh‐106 electrons, I‐125 plaque radiation penetrates deeper than that of Ru‐106/Rh‐106 plaque and can be used to treat tumors with thicknesses up to 10 mm. Ru‐106/Rh‐106 and I‐125 plaques are both low to intermediate dose‐rate sources with a maximum dose rate of a few Gy/h. As a result, the typical treatment time with the low dose‐rate eye plaque brachytherapy is on the order of a few days.

**TABLE 1:** Seed isotopes used for eye plaque brachytherapy.

	Decay mode	Peak energy	Average energy	Half‐life [days]
Ru‐106/Rh‐106	*β* ^‐^	3.54 MeV	1.42 MeV	373.6
I‐125	EC	35.5 keV	28 keV	59.5
Pd‐103	EC		21 keV	17
Y‐90	*β* ^‐^	2.281 MeV	0.934 MeV	2.7

A novel high dose‐rate Yttrium‐90 (Y‐90) disc source has been recently developed by Liberty Vision, NH, USA, and is commercially available now.^7^ Y‐90 is a beta emitter with a maximum energy of 2.281 MeV, an average energy of 0.934 MeV, and a half‐life of 64.2 h. Unlike photons, electrons from this radioisotope and other beta emitters mentioned earlier have a limited range in tissue, and a fast dose falloff at the tumor boundary is expected. This high dose‐rate Y‐90 source can deliver up to 800 Gy/h at 1 mm from the seed surface in water, which makes it possible to reduce the treatment duration to a few minutes.

Monte Carlo (MC) simulation is a computational technique used to estimate the possible outcomes of random events. It was invented by John von Neumann and Stanislaw Ulam during World War II to assist decision‐making under uncertain conditions. Monte Carlo Simulations possess several unique advantages over other analytic methods: (1) it can predict the outcomes of very complicated physical processes, (2) the results can be very accurate, and (3) it can also be used to analyze or calculate the correlation between inputs and outcomes. In medical physics applications, Monte Carlo Simulations have been used to model the particle transport and interactions of particles with matter based on the probability distribution functions determined by the physics processes involved. The energy transferred from the particles to matter along their traces are recorded and used for the final dose distribution calculation.

In this study, Monte Carlo methods were used to independently verify the dosimetry data of the new Y‐90 source. The simulated dose distribution of this source was compared with the results of the experimental measurements. This study could be used to establish guidelines for treatment planning of the novel high dose‐rate Y‐90 source in episcleral brachytherapy.

## 2 METHOD

### 2.1 The new Y‐90 source

**FIGURE 2:**
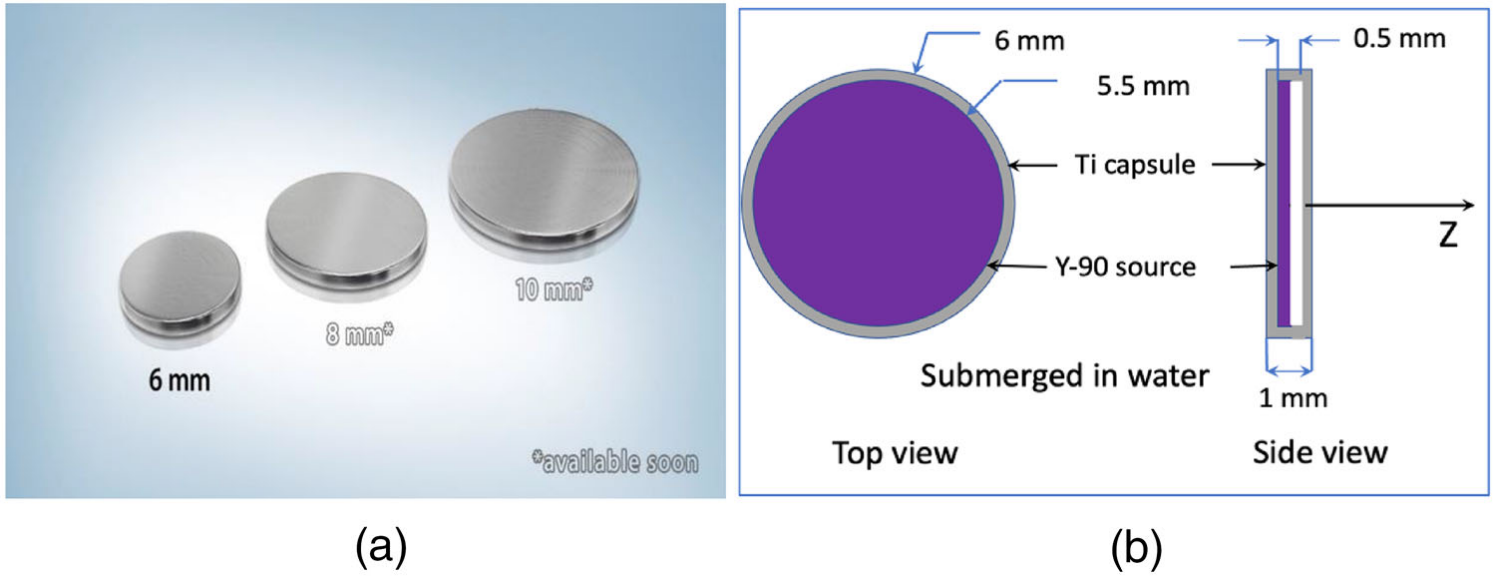
(a) Commercially available Y‐90 disc sources with diameters of 6 mm, 8 mm, and 10 mm. (b) Detailed structure of the 6 mm diameter disc source.

Figure 2a shows this new Y‐90 disc of various (6‐mm, 8‐mm, and 10‐mm) diameters, and Figure 2b illustrates the detailed structure of the 6‐mm disk. The range of 2.281 MeV (the peak energy of electrons from Y‐90 beta decay) electrons is 11.3 mm in water or 63% of the range (18.0 mm) for the 3.54 MeV (the peak energy of electrons from Ru‐106/Rh‐106 beta decay) electrons. The range of 0.934 MeV (the average energy of electrons from Y‐90 beta decay) electrons is 4.0 mm in water, or 60% of the range (6.64 mm) for the 1.42 MeV (the average energy of electrons from Ru‐106/Rh‐106 beta decay) electrons. Since Ru‐106/Rh‐106 is suitable for treating 5 mm size tumors, this Y‐90 source should be suitable for treating tumors with thickness up to ∼5 mm × 60% = 3 mm, which will be validated in this study.

### 2.2 Monte carlo simulations

There are several general‐purpose MC codes that can be used for brachytherapy dose calculations, including EGS4,^8^ EGSnrc,^9^ MCNP,^10^ and GEANT4.^11^ In this study, we chose GATE^12^ as our MC simulation tool. GATE is a GEANT4 based MC simulation tool developed by the international OpenGATE collaboration. GATE provides a powerful user interface so that the users can take full advantage of the GEANT4 simulation toolkit including well‐validated physics models, sophisticated geometry description, and powerful visualization and 3D rendering tools.

For this study, we downloaded and installed the GATE package in a Linux‐based workstation running the Ubuntu system. The physics processes that were simulated include Compton, Photoelectric, and pair production for photon particles; Electron ionization, Continuous Slowing Down Approximation (CSDA) model, and Bremsstrahlung for electrons and positrons. The PENELOPE (PENetration and Energy LOss of Positrons and Electrons) physics models of GEANT4 were chosen for the electromagnetic processes. This simulation work consisted of mainly two parts: (1) Validation of the MC codes, and (2) Calculation of the dose distribution for the Y‐90 disc source.

### 2.3 Validation of the Monte Carlo code with published data

**FIGURE 3:**
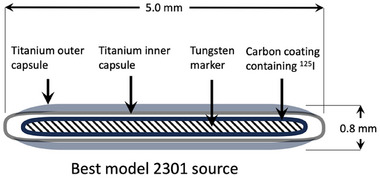
Dimensions of the Best Model 2301 I‐125 source.

Before any meaningful simulations can be performed, a solid validation of the code must be done to confirm that the GEATN4 software was properly installed and the simulation parameters of the code were set correctly. The code was first validated by simulating an I‐125 brachytherapy seed, the Best Model 2301 source, which was characterized in detail in the Update of AAPM TG43 report.^13^ Dimensions of this seed are shown in Figure 3.

**FIGURE 4:**
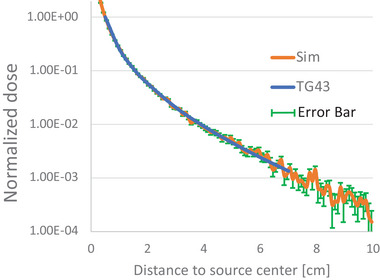
Comparison of the transverse‐plane dose distribution as a function of distance from the source for the Best Model 2301 I‐125 seed. “TG43”: data from Table XV of TG43.^13^ “Sim”: the Monte Carlo simulation results. Both curves were normalized to the 5 mm distance from the source.

Geometries of the I‐125 source and the water phantom were modeled in the GEANT4 simulation software with the source located at the center of the water phantom. About 10^7^ simulation histories were performed to obtain the transverse‐plane dose distribution as a function of distance from the source. Results of this validation are shown in Figure 4, where the blue curve plots the data from Table XV of TG43 report, and the orange curve from the Monte Carlo simulation. Both curves were normalized to the 5 mm distance from the source. The transverse‐plane dose distribution of the I‐125 source simulated from this study agreed very well with that reported in TG43 up to 7 cm, the maximal distance reported in Table XV of TG43.^13^

**FIGURE 5:**
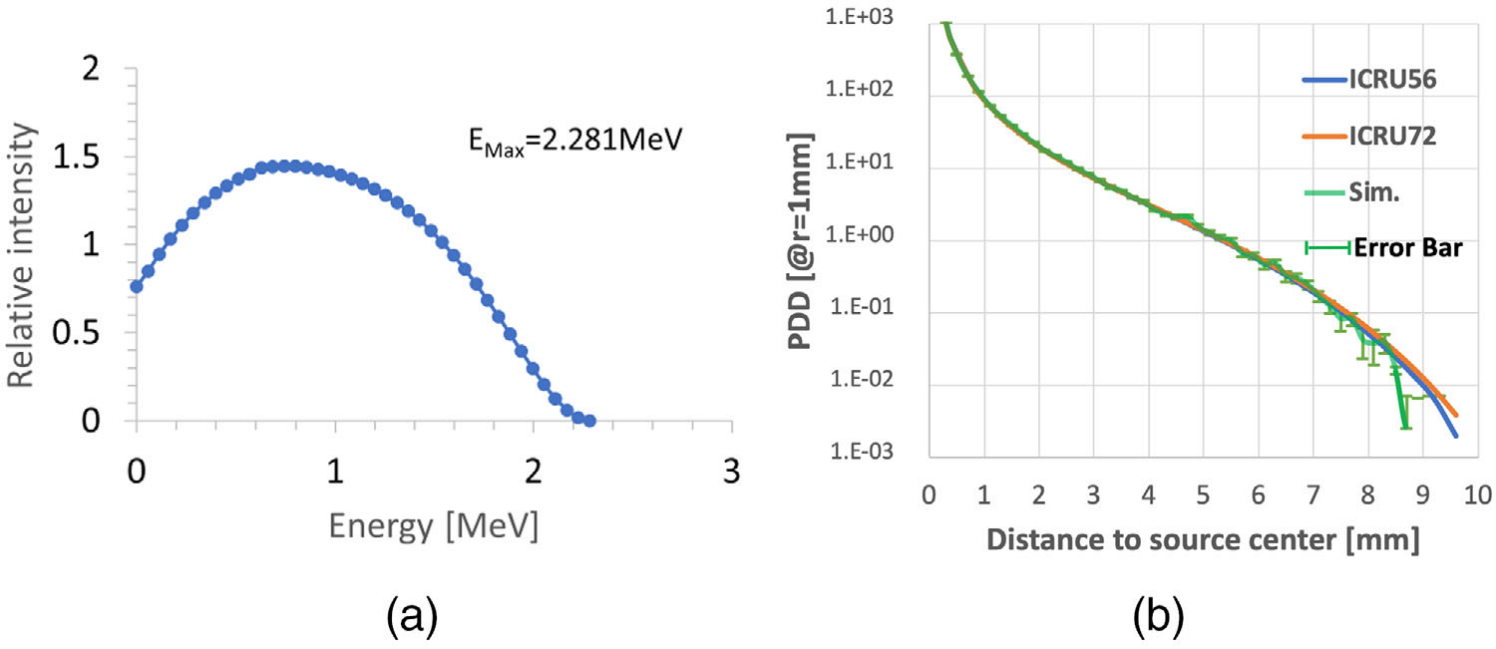
(a): Electron energy spectrum of an isotropic Y‐90 point source. (b): Comparison of the point‐source dose distribution as a function of distance from the source, from the simulation for 10^7^ histories, Table C.10 of ICRU 56^14^ and Table 4.3 of ICRU 72,^15^ as a function of distance from the source.

The code was then revised to simulate the dose distribution of an isotropic Y‐90 point source as described in ICRU 56^14^ and ICRU 72.^15^ The electron emission spectrum of this Y‐90 point source in Table A.3 of ICRU 72 was adopted as the Geant4's radioactive source energy spectrum for this simulation. As shown in Figure 5a, the electron energy spectrum of the Y‐90 source has a maximal electron energy of 2.281 MeV and peaks at an energy of about 0.74 MeV.

With this spectrum, we were able to simulate the dose distribution of this Y‐90 isotropic point source. The initial energy of each sample electron was randomly determined according to the emission spectrum in Figure 5a, and the initial emission direction was also randomly sampled from a uniform spherical probability density function. The interactions of all initial and secondary particles were governed by the material properties and the probability functions of involved physics processes including photoelectric, Compton, pair‐production, electron ionization, bremsstrahlung, and CSDA model.

The simulation results were compared with that in ICRU 56^14^ and ICRU 72.^15^ Figure 5b shows the point‐source dose distribution from the simulation for 10^7^ histories, Table C.10 of ICRU 56^14^ and Table 4.3 of ICRU 72,^15^ as a function of distance from the source. All three curves in Figure 5b agree very well up to 7 mm, which validated the simulations performed in this study.

### 2.4 Monte Carlo simulations and experimental measurements of the Y‐90 disc source

**FIGURE 6:**
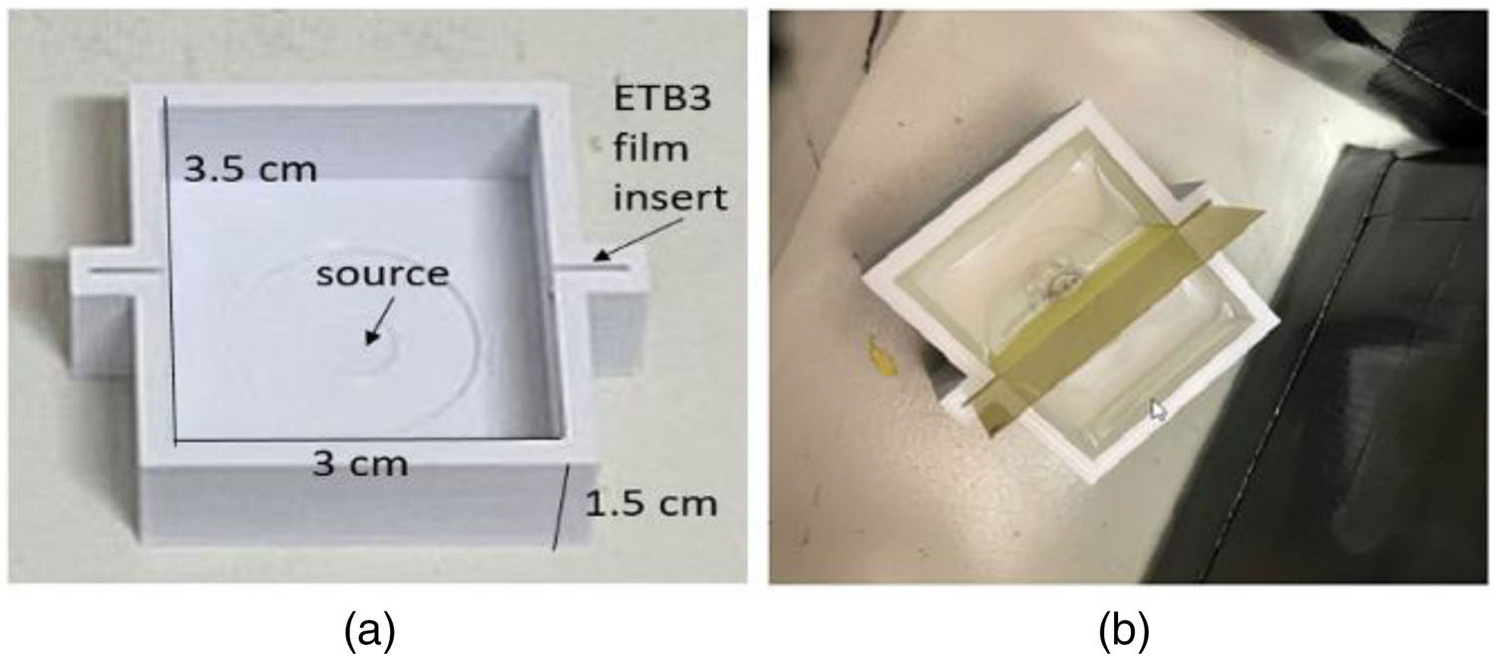
(a) Schematic design of the 3 cm × 3.5 cm × 1.5 cm 3D‐printed phantom for depth‐dose measurements. (b) Experimental setup filled with water.

The experimental measurements on the dose distribution of the Y‐90 disc source with film dosimetry and customized phantoms (Figure 6) have been briefly presented in our conference proceedings.^16^, ^20^ Calibration of the films (0–10 Gy) was perfomed using the 6‐MV photon beam of a Varian Truebeam LINAC. All films were processed and analyzed following the same protocol as described in the reference.^21^ Phantoms that provide a water environment and accurate film positioning were designed on TinkerCAD and fabricated with a low‐cost desktop 3D printer (Ender 3 v2) using Polylactic Acid (PLA) filament and a 0.4 mm nozzle. The phantom shown in Figure 6a is an example of the central axis depth dose measurements. The phantom is a well‐like structure with handles on the side. During measurements, the phantom is filled with water (Figure 6b). The Y‐90 disc source is placed at the center of the bottom plate with its surface flush to the plate. The film was then inserted vertically through the guiding slots on the handles until it reached the source surface. After irradiation, films were scanned on the Epson‐10000XL flat‐bed scanner after 24 h and analyzed via the RIT V6.8 Film software package. The PDD curve was then obtained by plotting the doses on the film along the source central axis and normalized to the reference point, 1 mm above the source surface. A separate publication featuring the experimental study is in preparation.

**FIGURE 7:**
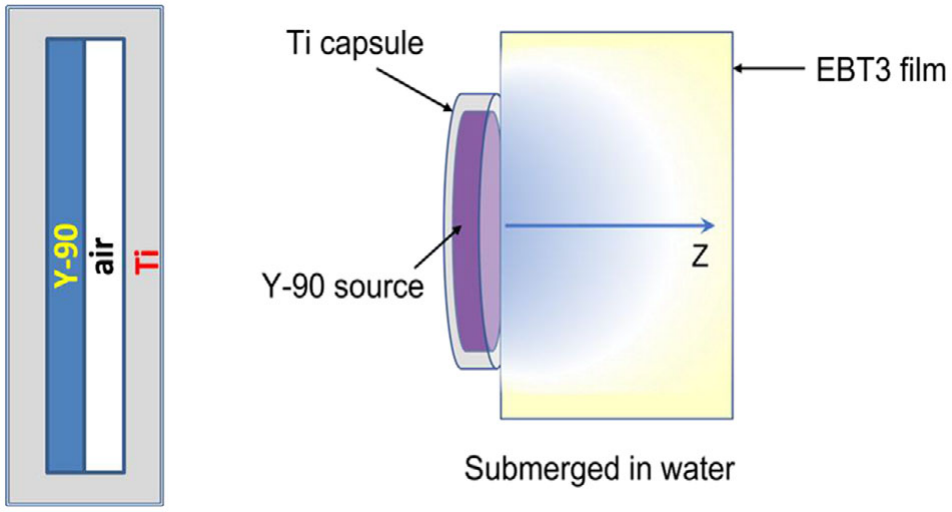
Schematic view of the Y‐90 simulation of measurement.

The geometry of the disk source as shown in Figure 2 was modeled in the simulation, along with the EBT‐3 film used for dose measurements. The simulation world, a20cm×20cm×20cm cube of air was first created, in which a cylinder (10 cm in diameter and 10 cm in height) of water phantom was generated. The simulation model as shown in Figure 7 was then constructed based on the exact experimental setup for comparison. In GEANT4, the initial location of each sample was randomly selected within the source geometry with an equal probability. The initial energy and emission direction, and following interactions were the same as the simulation for the Y‐90 point source described in the previous section. 2 × 10^7^ histories were used in each simulation. For each history, the dose deposited at each voxel along its trace was recorded and cumulated to obtain the final 3D dose distribution.

## 3 RESULTS

**FIGURE 8:**
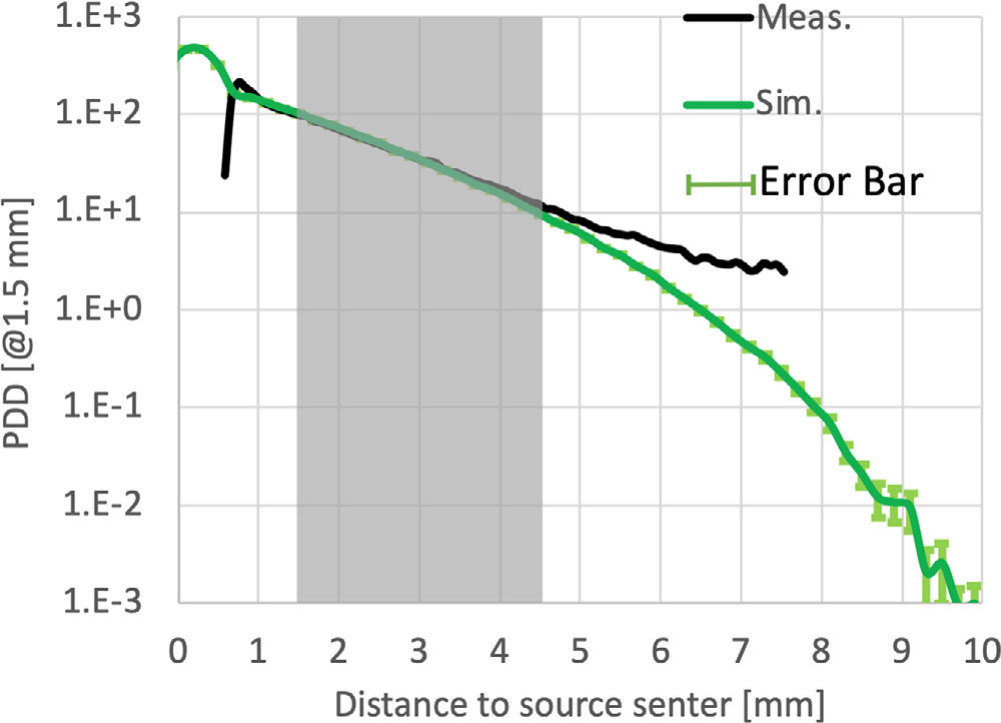
Comparison of the measured and Monte Carlo (MC) simulated PDD curves. In this plot, the center of the disk source is located at 0 mm, and the surface of the source package is at 0.5 mm. Black: measurements using EBT‐3 film. Green: MC simulation for the Palmer EBT‐3 film. Both PDD curves were normalized to the reference point located at 1.5 mm from the source center or 1 mm from the package surface.

The measured PDD along the central axis of the source and the calculated PDD from MC simulations are plotted and compared in Figure 8. Both PDD curves were normalized to the reference point located at 1 mm from the source surface along the central axis, which corresponds to 1.5 mm from the source center along the central axis. The two PDD curves agree well up to 4.0 mm from the surface with the maximal relative difference ($\frac{\mathrm{MC}-\mathrm{Measured}}{\mathrm{Measured}}$) being less than 10%. Above 4 mm, these two PDD curves deviate from each other. Note that there is a buildup region in the measured but not the simulated curve. We believe this buildup region is an artifact since it is located at the edge of the film, where the sensitivity had been compromised from the cutting of the film.

The box shaded in gray in Figure 8 indicates the clinical range of the PDD that can be used for treatment planning. If we define the treatment depth of this new source as where the dose drops to 20% of the dose at the reference point, the treatment depth is about ∼3.2 mm from the surface of the source package.

**FIGURE 9:**
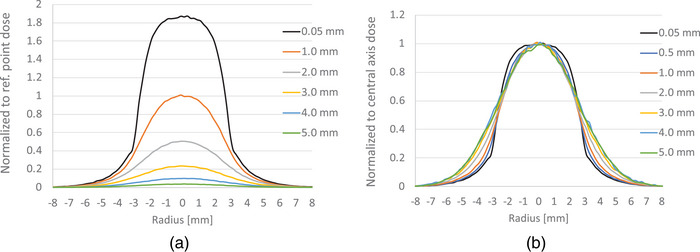
(a) Dose distributions at various planes parallel to the source surface, normalized to the reference point (1 mm from the source surface along the central axis). The numbers in the legends represent the distances to the seed surface. (b) Same distributions as (a) but normalized to the center dose of each plane.

Figure 9a shows the 2D dose distributions of the source on a few planes parallel to the seed surface, and Figure 9b illustrates the same distribution as Figure 9a but normalized to the center doses of each plane. Table 2 lists the fall‐off of dose profiles at different depths in mm for 90%−10% and 80%−20% dose (relative to the central axis dose of each plane), and lateral treatment range for 80%−80% and 90%−90% dose. It can be observed from Figure 9b and Table 2 that the lateral dose coverage depends on the normalized percent isodose chosen for the coverage. If 80% dose is selected for coverage, the lateral dose coverage is maximal (∼4.5 mm) at the plane next to the source surface, and gradually decreases with the increasing distance, approaching 3.5 mm when the plane is 5 mm away from the seed surface. The lateral dose coverage is about the same (∼5 mm) for all planes if 60% isodose is selected.

**TABLE 2:** Fall‐off of the dose profile in mm at various depths for 90%−10% and 80%−20% dose and lateral treatment range for 80%−80% and 90%−90% dose. All % doses are normalized to the central axis dose of each plane.

Depth from the surface (mm)	Dose fall off distance (mm)		Range (mm)		
	90%−10%	80%−20%	90%−90%	80%−80%	60%−60%
0.05	2.2	1.1	3.7	4.2	4.6
1	3.2	1.9	2.5	3.5	4.7
2	3.8	2.4	2.3	3.3	4.8
3	4.1	2.6	2.3	3.4	5.0
4	4.1	2.8	2.5	3.4	5.2

The dose at the reference point of 1 mm above seed surface center was 0.0294 Gy after 10^8^ of histories, corresponding to $\;100\times 0.0294\;cGy/\frac{10^{8}\;dist}{3.7\times 10^{7}dist/\text{mCi-s}}=\;$ 1.09 cGy/mCi‐s. If a 20 mCi seed is used for treatment, it would have a dose rate of $20\;mCi\times \frac{1.09\frac{cGy}{s}}{mCi}\times 60\;s/min=\;$13.1 Gy/min at the reference point. In comparison, the dose rate from film measurements was 0.604 Gy/min at 1.02 mm for an activity of 0.96 mCi, corresponding to 12.58 Gy/min after being scaled to the 20 mCi activity. In addition, the reported dose rate from the vendor is 16.2 Gy/min for a source of the same activity.

The max strength of the Liberty Vision new source is ∼80 mCi for shipment and the max strength used for treatment is 20 mCi. With the measured and simulated dose rate, the treatment time for a prescription dose of 10 Gy at depths 1, 2, 3, and 4 mm from the source surface delivered by a 20 mCi source could be calculated and are tabulated in Table 3. In Table 3, the second column is the treatment time calculated using the dose rate from the Monte Carlo simulations, and the last column lists treatment time calculated from vendor‐supplied dose rate data. Note that the vendor only provides dose rate data for three of the eight distances in Table 3. It is observed from Table 3 that for a prescription dose of 10 Gy, the dose delivery could be completed in less than 4 minutes if the prescription depth is 3 mm or less. To be mentioned, a prescription dose of 10 Gy is selected here for the convenience of calculation. Based on a recent study from Finger et al, the prescription dose ranges from 22 to 30 Gy at varied source activities and prescription depths.^22^

**TABLE 3:** Treatment time for a prescription dose of 10 Gy at depths 1, 2, 3, and 4 mm from the source surface, delivered by a 20 mCi source. The second column is the treatment time calculated using the dose rate from the Monte Carlo simulations. Among these distances, the treatment times (min) calculated from the vendor‐supplied dose rate data for three (0.6 mm, 1 mm, and 2.6 mm) distances are listed in the third column.

Distance from the seed surface (mm)	Treatment time based on our simulations (min)	Treatment time based on vendor data (min)
0.6	0.6	0.46
1	0.76	0.617
1.5	1.06	
2	1.51	
2.6	2.36	2.08
3	3.25	
3.5	4.75	
4	7.6	

## 4 DISCUSSION

In this study, we used Monte Carlo methods to independently verify the dosimetry data of the new Y‐90 source for episcleral brachytherapy. The central axis PDD curve from the simulations was compared with that from the experimental measurements. 2D dose distributions of this source for planes at different depths were calculated to determine the range of dose coverage at different treatment depths. Treatment times for a maximum 20 mCi source were also calculated for a nominal prescription dose of 10 Gy to different treatment depths and compared with the calculations based on vendor‐supplied data.

The PDD curve from MC simulations agreed well with the measurements up to 4.0 mm (<±10%) from the source surface but deviated beyond that depth. A similar study by Rogers et al^17^ shows a smaller difference between the measurement and MC result, 6% up to 5 mm above their seed surface. The larger deviation in our study may be due to the different structure of our seed, which has an extra 0.25 mm thick Ti capsule; but the measurement uncertainties in the low dose region may be the main reason for this deviation. This deviation does not affect our analysis regarding the recommended treatment depth (∼ 3.2 mm) of the source, where the dose drops to 20% of the dose at the reference point of the PDD curve. Note that this recommended treatment depth of 3.2 mm is consistent with that (∼ 5 mm) of Ru‐106/Rh‐106 considering the difference in electron energy. That is, since the ratios of their average energy electron ranges and their peak energy electron ranges are both *R*_Y‐90_/*R*_Ru‐106/Rh‐106_ = 62%, the scaled treatment depth for Y‐90 should be around 5×0.62=3.1mm, which is very close to 3.2 mm determined from this study. If we define the treatment depth to where the dose drops to 10%, the depth is ∼ 4 mm. As the Ru‐106/Rh‐106 seeds have been used to treat tumors up to 11 mm in depth,^18^, ^19^ it is reasonable to assume that this Y‐90 source can be used to treat tumors up to 7 mm in depth.

It is also found that the seed has a fast dose fall‐off not only along the axis direction but also in the transverse direction. Currently, only the 6 mm‐diameter source is available commercially. The 8 mm‐ and 10 mm‐diameter seeds are expected to come soon but the lateral ranges for tumor coverage have not been reported for these different disc sizes. For the 6 mm‐diameter disk, the lateral ranges of PTV that can be covered is ∼ 2.3 and ∼3.3 mm if the treatment plan is normalized to 90% and 80% respectively of the dose at the reference point, as shown in Figure 9 and Table 2. This range is much smaller (40%−60%) than the actual diameter of the disk. Therefore, there might be a high risk of under‐dosing the periphery of the tumor if the difference between the disk size and the actual range of dose coverage is not considered when planning the treatment.

The simulated dose rate at the reference point, 1 mm above the surface on the central axis of the source, for a 20 mCi seed was 13.1 Gy/min and this result agreed well (∼5% higher) with the measured 12.5 Gy/min at 1.02 mm for a source of the same activity. The dose rate specified by the vendor at the same location, on the other hand, is 16.2 Gy/min for the same activity, which is 24% higher than our results. Further investigation of this difference is warranted. In any case, we can estimate the ballpark treatment time (Table 3) needed for a conventional treatment using this source. As shown in Table 3, the treatment for a 10 Gy prescription at 2 mm depth using this new Y‐90 source can be finished in 1.5 minutes for a 20 mCi source.

There are a few limitations with this new Y‐90 source. First, only 6‐mm diameter source is available at this stage, which limits the applicability of this high‐dose‐rate treatment to larger lesions. In addition, the short half‐life (2.6 days) of Y‐90 may lead to inconveniences in patient scheduling. A one‐hour delay in the procedure results in about a one‐percent decrease in source strength. If there is an unexpected delay of a few days, the ordered source may become unusable due to its low activity, necessitating a new order. For instance, the lowest activity reported in clinical use is 8.7 mCi.^22^ Let's consider a scenario where a maximum strength of 20 mCi was ordered for the treatment day. In this case, a delay of only 3.2 days in the treatment would cause the source to reach the lowest activity currently reported in clinical use. Another challenge of this seed in clinical applications may come from the storage requirement. Although the electrons can be easily shielded, the generated bremsstrahlung photons can penetrate much farther and will require a storage bucket with a thicker wall. The high dose rate also requires an effective way to place the source to the tumor location quickly so that the dose to the normal tissues and to the staff can be minimized. Finally, the 3.2 mm treatment depth, which is significantly smaller compared to that of Ru‐106/Rh‐106 seeds, might also limit the clinical application of this seed.

## 5 CONCLUSIONS

In conclusion, the clinical parameters of this new Y‐90 source required for the treatment planning of episcleral brachytherapy have been investigated in this study using Monte Carlo simulations. The PDD curve from Monte Carlo simulations agrees well with experimental measurements within 4.0 mm (< ± 10%) above the seed surface but deviates beyond 4.0 mm. Measurement uncertainties in the low‐dose region may be the main reason for the deviation. This deviation does not affect our analysis regarding the clinical application of the seed, which is recommended for tumors less than 3.2 mm thick. It is also found that the lateral coverage is much smaller than the diameter of the source disk. The dose rate at the reference point is 1.09 cGy/mCi‐s so a treatment of 10 Gy prescription dose at 3 mm depth can be finished in less than 4 minutes. This simulation work, along with the experimental measurements to be published, laid a solid foundation for characterizing the full dosimetry parameters of this source for clinical applications.

## AUTHOR CONTRIBUTIONS

All authors have significant contributions to this paper. Xiangyun Chang carried out the majority of Monte Carlo simulations and data analysis of this work. Lyu Huang performed the dose measurements of the source. Jian Liu installed and configured the GATE software and provided many useful tips for running the simulation codes. Yijian Cao had participated in all discussions and made many useful clinical suggestions.Jenghwa Chang, the corresponding author, supervised all research activities,  guided discussions, and oversaw the manuscript's preparation. Previous Presentations: Part of this work was presented at the AAPM 64th Annual Meeting, Washington DC, July 10–July 14, 2022.

## CONFLICT OF INTEREST STATEMENT

The authors declare no conflicts of interest.
